# Endoscopic Ultrasound for the Detection of Left Atrial Appendage Thrombus: A Useful Technique in Patients with Transesophageal Echocardiography Contraindication

**DOI:** 10.1155/2016/7387946

**Published:** 2016-08-25

**Authors:** Manuel Marina-Breysse, Alfonso Jurado-Román, Bartolomé López-Viedma, Jesús Piqueras-Flores, María T. López-Lluva

**Affiliations:** ^1^Cardiology Department, University General Hospital of Ciudad Real, Ciudad Real, Spain; ^2^Myocardial Pathophysiology Area, Centro Nacional de Investigaciones Cardiovasculares [Spanish National Centre for Cardiovascular Research] Carlos III (CNIC), Madrid, Spain; ^3^Interventional Cardiology Department, University General Hospital of Ciudad Real, Ciudad Real, Spain; ^4^Digestive Department, University General Hospital of Ciudad Real, Ciudad Real, Spain

## Abstract

Endoscopic ultrasound is a diagnostic and therapeutic technique used in specialized centers for patients that have undergone digestive procedures. This technique enables highly precise real-time imaging of the digestive tract wall and surrounding organs. Endoscopic ultrasound is also useful in patients with cardiovascular diseases such as atrial fibrillation. In patients with contraindication for transesophageal echocardiography due to high risk of esophageal bleeding or complications that may require immediate intervention, endoscopic ultrasound may be a safer option for visualizing atrial chambers to rule out the presence of left atrial appendage thrombi before cardioversion.

We present a 75-year-old woman admitted with symptomatic persistent atrial fibrillation (AF) with rapid ventricular rate. CHA2DS2-VASc risk score was 5 considering hypertension, age, gender, and diabetes. HAS-BLED score was 4 due to type C-derived hepatitis with cirrhosis and esophageal varices. As we first decided rhythm control strategy, ruling out the presence of left atrial appendage (LAA) thrombi was mandatory before cardioversion. However, patient's history of esophageal varices was a relative contraindication for transesophageal echocardiography (TEE) [1]. Therefore, we decided to use an endoscopic ultrasound approach as described previously [2, 3], which indeed might have provided an additional support in case of esophageal bleeding. With a Pentax EG-3870UTK endoscope coupled to a Hitachi HI Vision Avius console (ultrasound beam frequency at 7.5 MHz), endoscopic ultrasound echocardiography was performed without complications. Video-endoscopic imaging provided direct visualization of the esophageal varices (Figure 1) and optimal visualization of the LAA which had a thrombus inside (Figure 2). Therefore, anticoagulation and rate control strategy to relieve symptoms were finally decided. One month later, the patient was asymptomatic and free from thromboembolic or hemorrhagic events.

Endoscopic ultrasound is a diagnostic and therapeutic technique. Most specialized centers use it for patients that have undergone digestive procedures such as gastrointestinal tumor staging, where endoscopic ultrasound has provided a major breakthrough for characterizing such tumors and distinguished between intramural and extramural lesions. Endoscopic ultrasound imaging is usually performed by positioning the distal ultrasound transducer (frequencies ranging from 5 to 12 MHz) adjacent to the lesion of interest. This technique enables highly precise real-time imaging of the digestive tract wall and surrounding organs. The key point is that these endoscopic probes are also useful in patients with cardiovascular diseases such as AF. In spite of being at that time a monoplanar device, endoscopic ultrasound has a high quality 2D resolution, color Doppler, and pulsed Doppler.

Endoscopic ultrasound may be a safer option for visualizing atrial chambers when the patient is at high risk of esophageal bleeding or complications that may require immediate intervention [4] as well as when patients have functional or anatomic variations in the hypopharynx/upper esophagus [2]. Although endoscopic ultrasound is generally safe, complications may occur secondary to sedation, therapeutic interventions, or advancement of endoscopic ultrasound. Rare complications may also occur with endoscopic ultrasound—incidence of esophageal perforation is 0.06%—with the advantage of being immediately identified during the procedure, and some of them—esophageal bleeding included—could be treated at the same time [4]. Clinical trials support its use in gastrointestinal hemorrhage due to esophageal varices [5] and the utility of this technique to assess the presence of LAA thrombi has been described in this report. This report opens a new field for further studies that would evaluate if endoscopic ultrasound can be a safer technique to rule out the presence of left atrial appendage thrombi before cardioversion when transesophageal echocardiography is contraindicated.

## Figures and Tables

**Figure 1:**
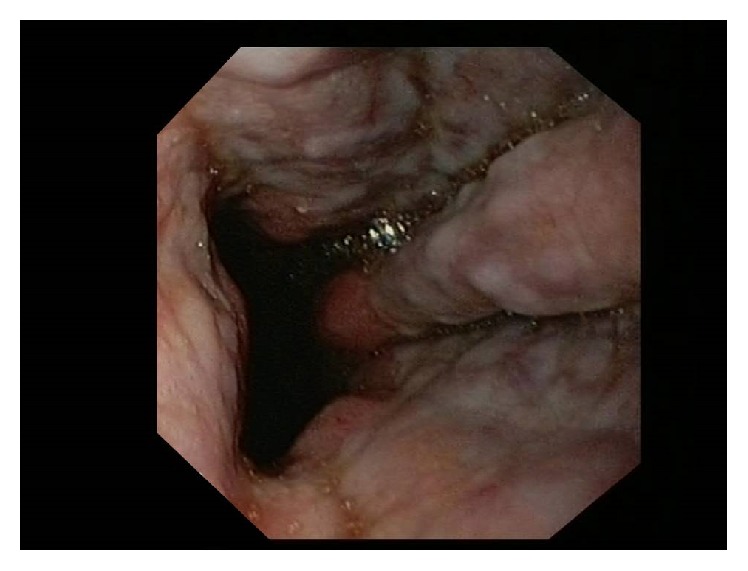
Grade III esophageal varices visualized by endoscopic ultrasound technique.

**Figure 2:**
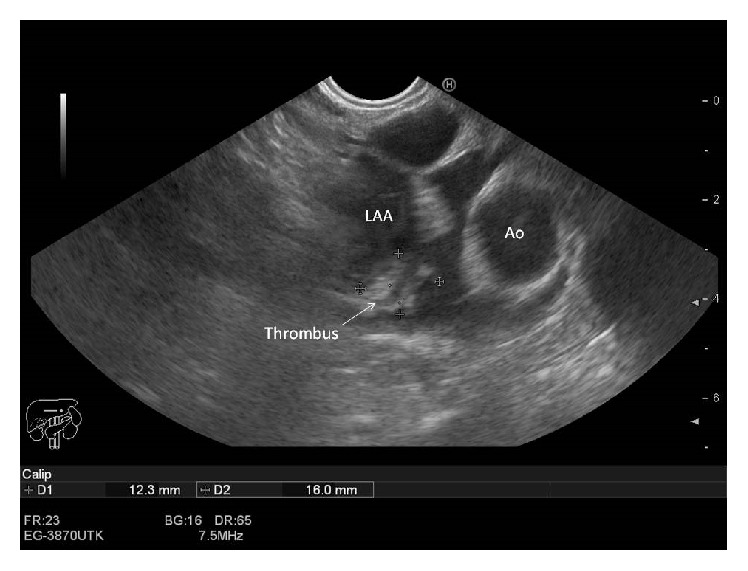
Thrombus detection in the left atrial appendage using endoscopic ultrasound technique. LAA: left atrial appendage; Ao: aortic root.

## Abbreviations

AF: Atrial fibrillation
LAA: Left atrial appendage
TEE: Transesophageal echocardiography
EUS: Endoscopic ultrasound.

## Additional Points

*Key Teaching Points*. Endoscopic ultrasound may be a safer technique to rule out the presence of left atrial appendage thrombi before cardioversion when the patient is at high risk of esophageal bleeding or transesophageal echocardiography is contraindicated.
